# Global Considerations in Hierarchical Clustering Reveal Meaningful Patterns in Data

**DOI:** 10.1371/journal.pone.0002247

**Published:** 2008-05-21

**Authors:** Roy Varshavsky, David Horn, Michal Linial

**Affiliations:** 1 School of Computer Science and Engineering, The Hebrew University of Jerusalem, Jerusalem, Israel; 2 School of Physics and Astronomy, Tel Aviv University, Tel Aviv, Israel; 3 Deptartment of Biological Chemistry, Institute of Life Sciences, The Hebrew University of Jerusalem, Jerusalem, Israel; University of Michigan, United States of America

## Abstract

**Background:**

A hierarchy, characterized by tree-like relationships, is a natural method of organizing data in various domains. When considering an unsupervised machine learning routine, such as clustering, a bottom-up hierarchical (BU, agglomerative) algorithm is used as a default and is often the only method applied.

**Methodology/Principal Findings:**

We show that hierarchical clustering that involve global considerations, such as top-down (TD, divisive), or glocal (global-local) algorithms are better suited to reveal meaningful patterns in the data. This is demonstrated, by testing the correspondence between the results of several algorithms (TD, glocal and BU) and the correct annotations provided by experts. The correspondence was tested in multiple domains including gene expression experiments, stock trade records and functional protein families. The performance of each of the algorithms is evaluated by statistical criteria that are assigned to clusters (nodes of the hierarchy tree) based on expert-labeled data. Whereas TD algorithms perform better on global patterns, BU algorithms perform well and are advantageous when finer granularity of the data is sought. In addition, a novel TD algorithm that is based on genuine density of the data points is presented and is shown to outperform other divisive and agglomerative methods. Application of the algorithm to more than 500 protein sequences belonging to ion-channels illustrates the potential of the method for inferring overlooked functional annotations. ClustTree, a graphical Matlab toolbox for applying various hierarchical clustering algorithms and testing their quality is made available.

**Conclusions:**

Although currently rarely used, global approaches, in particular, TD or glocal algorithms, should be considered in the exploratory process of clustering. In general, applying unsupervised clustering methods can leverage the quality of manually-created mapping of proteins families. As demonstrated, it can also provide insights in erroneous and missed annotations.

## Introduction

Clustering is a common unsupervised machine learning procedure. It is often used for preprocessing, and usually provides a general overview, especially when dealing with large datasets. Its applications range from astronomy to economics, psychology marketing, text mining and other areas. Recent advances in genomic biology high-throughput techniques have led to a growing need for efficient and powerful clustering algorithms [1]. For instance, in large-scale gene expression data, clustering algorithms are useful in the diagnosis of different samples (e.g., diseased and healthy patients, labeling of tissues by disease subtype), as well as for their ability to reveal functional classes of genes among the thousands often used in experimental settings [1], [2].

Clustering algorithms are often classified as either nonhierarchical (partitioning) or hierarchical. The former define a complete partition of the data (for comprehensive reviews see [1], [3], [4]). Because they suggest multiple levels of organization, hierarchical algorithms are best suited for describing data that have some inherent breakdown resolution. Organizing complex arrangements into hierarchies is a common technique in many fields, such as grammar description in computational linguistics, industrial organization (NAICS - The North American Industry Classification), object oriented programming, biological taxonomy and evolutionary organization of proteins, genes or species. Hierarchical clustering has been successfully applied to protein sequences, chemical entities, 3D structural information and protein catalytic activities [5].

The outcomes of hierarchical algorithms can be represented as a tree, where each node branches into two (a ‘binary tree’) or more nodes. Ideally, the tree has some underlying basis; for instance, sub-industry breakdown, or protein families that reflect evolutionary diversification. In any case, it can represent many clustering solutions corresponding to different groupings of nodes. A collection of nodes may be viewed as natural cuts in the tree. Some of the clustering possibilities may match an expert's view. Other clusters may correspond to a pattern exposing the nesting in the data (sub-classes) which a given expert may not have been aware of. In fact, this is the rationale behind the clustering approach; namely, finding new internal patterns in the data. Since hierarchical clustering provides alternative clustering possibilities, it is usually considered as a richer tool than the single, nonhierarchical, clustering solution.

Hierarchical methods can be further divided into Bottom-Up (BU, agglomerative) and Top-Down (TD, divisive) types [3], [4], [6]. BU algorithms start with each instance as a cluster and repeatedly merge clusters until a unified cluster is formed. They are popular in genomics (gene expression [1], and proteomics [7], and have been implemented in resources such as ClusTr [8] and ProtoNet [9]. TD methods work in the opposite direction and are rarely used for these types of data. Although most tutorials present the two strategies, and some works have recently suggested ways to combine them [10], BU algorithms are significantly more popular than TD algorithms. A survey of all articles published in PLoS in the last two years (years 2006–2007) shows that out of 86 publications that apply hierarchical clustering to analyze data, only 3 do not utilize the standard BU approach. This significant bias toward the BU approach is mostly due to its availability in software packages [2], [11] and intuitive appeal. Furthermore, the reliability at the beginning of the clustering process is evident and no assumption on any statistical model in the data is required. These reasons probably led most researchers to neglect the TD approach as a potential approach for unlabeled data.

Although less popular, several recent TD algorithms have been found to be highly efficient, especially in document classification problems. One such example is the Bisecting K-Means algorithm, based on the divide-and-conquer scheme of repeated K-means (K = 2). It outperforms both standard K-Means and agglomerative clustering [12], and is computationally efficient [13]. It suffers, however, from the usual problems of the K-means approach; namely a bias toward spherical clusters and a dependency on initial conditions. The second such example is Principal Direction Divisive Partitioning (PDDP), which is based on repeated divisions of instances according to the sign of their projection on the first principal component [14]. PDDP outperforms the bisecting K-Means algorithm in quality and stability [15] and will thus be used here as a benchmark for a state-of-the-art TD algorithm.

This paper examines the advantages of involving global approaches in clustering, and demonstrates that they can generate meaningful results near the top of the hierarchy tree. It tests and compares different approaches on three extensively studied benchmarks. The TD algorithms succeed better in capturing the expert assignment as compared to the state-of the-art BU clustering methods. Moreover, a novel TD algorithm, called TDQC (Top-Down Quantum Clustering) is then presented and shown to outperform other algorithms. TDQC is based on an algorithm which has been applied to gene expression datasets [16] that were initially processed by SVD. In addition, an intermediate approach, named ‘glocal’, which is a BU based clustering with global consideration, is suggested to handle datasets represented by distances (and not in their feature space).

The datasets and the algorithm are described in the next section. After the comparative study of various TD and BU algorithms on the three benchmarks we apply them to a functionally coherent protein dataset. The application of TD to a protein set leads to biological insights that can reveal intriguing patterns in the data. ClusTree, a new validation and visualization tool that was used to compare the performance of the different hierarchical classification methods is provided.

## Materials and Methods

### Datasets

Various clustering methods are applied to four different types of datasets. These sets are the basis for a comparative analysis of previous studies and existing algorithms. Two of the sets are known benchmarks of gene-expression experiments. The third set is a known stock-market dataset, and forth is a biological dataset of ion-channel proteins.

**Cell Cycle genes** Spellman *et al*. identified 798 genes as cell cycle regulated in the yeast *Saccharomyces cerevisiae* and catalogued them into five classes that correspond to different stages of the yeast cell cycle (marked as M/G1, G1, S, G2 and M). Expression levels of those genes were recorded at 72 continuous time-points yielding a [798 genes×72 time-points] matrix (Table S3, supplementary material).

**Leukemia patients** The Golub *et al*. dataset has served as a benchmark for several clustering methods [17]–[19]. The experiment sampled 72 patients with two types of leukemia, ALL and AML. The ALL set is further divided into T-cell and B-cell leukemia and the AML set is divided into patients who underwent treatment and those who did not. For each patient, the expression levels of 7129 genes is reported. The clustering task is to find the four cancer groups within the 72 patients in a [72 patients×7129 genes] gene expression matrix (Table S4, supplementary material).

**Standard and Poor (S&P)** We used the stocks dataset of [20], who collected day-to-day fractional changes in the price of all stocks in the Standard and Poor's 500 list during the 273 trading days of one year. 487 of the stocks are divided in 10 different industry segmentations. The dataset is organized in a [487 stocks×273 trade days] matrix (Table S5, supplementary material).

**Ion Channel proteins.** The dataset is extracted from the SwissProt database (version 40.28). For the 614 proteins that are annotated as ‘ion channel activity’ (according to Gene Ontology, ID-5126), all-against-all BLAST E-values are recorded [21]. All E-values lower than 100 are kept in a matrix and E-values higher than 100 are limited to be 100. 518 of these proteins are annotated by the InterPro (http://www.ebi.ac.uk/interpro/, version 7.0) collection, thus resulting a [518 proteins×518 proteins] distances matrix. Only exclusive InterPro labels were considered. There are ∼40 exclusive InterPro labels that are associated with at least 2 proteins each. Several levels of granularity are associated with this protein set. The 3 group labels are ‘ligand-gated channel’, ‘voltage gated’ and ‘others’. These 3 classes describe a gross partition. This gross classification can be nested into 11 classes which can be further nested into 19 classes. The 3 resolution levels are considered *gross*, *medium* and *detailed* mapping (Tables S1, S2, supplementary material).

### The TDQC algorithm

**Figure 1:**
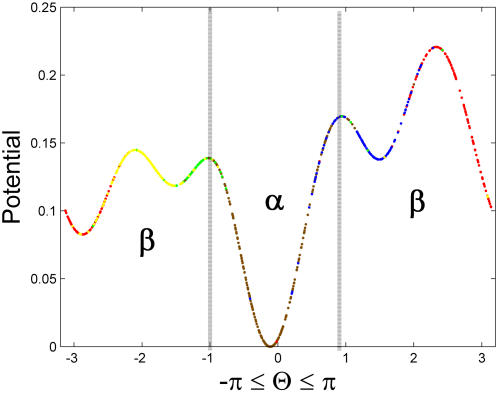
Potential values of the cell cycle dataset. Data were projected onto the two leading SVD components, and represented in terms of the angle in these coordinates. Dashed lines mark the partitioning of the dataset into two groups (α and β). For details see text. The color code represents Spellman's expert view for the 5 cell cycle phase (G1-brown, number of instances, S-green, S/G2- yellow, G2/M-red, M/G1-blue).

The TDQC algorithm is defined as follows:

0. Define original dataset (Number of sets = 1)
1. [Optional] Apply preprocessing to each set
2. Run QC (Quantum Clustering) on each set
3. Divide each set into two sets containing:
  - Instances belonging to the cluster with the global minimum (A in Fig. 1)
  - All the rest (B in Fig. 1)
4. Recursively go-to 1 for each set including more than 2 instances

### Preprocessing

In order to transform the data into a compressed, manageable and hopefully noise-free representation, it is recommended to use the Singular Value Decomposition (SVD) method. SVD represents any real matrix *X* of size *[nXm]* as a product *X = U*Σ*V^T^*, where *U* and *V* are orthonormal matrices and Σ is a diagonal matrix whose eigenvalues *s_i_* (singular values) appear in decreasing order. In this context, *n* is the number of instances (or elements), and *m* is the number of features (or attributes), describing each instance. The columns of *U* and *V* define two independent vector spaces. Rather than studying the resulting low-rank matrix *X′ = U*Σ*′V^T^* (by zeroing all singular values at locations *i>r*, one can compress the data into an *r*-dimensional space), we focus our attention on the *r* first columns of the unitary matrices *U* and *V*. It is within these vector spaces that we look for cluster structures [16], [22], [23]. Common wisdom is to choose *r* such that the first *r* eigenvalues explain most of the variance (above a certain threshold) of *XX^T^*. Other heuristics look for an ‘elbow’ in the singular values graph. Several other guidelines are discussed at length in [24], [25].

Following the experience of Latent Semantic Analysis (LSA), in computation linguistic [26], we define distances among the *r-*dimensional vectors in terms of cosines of the angles among them, as *d* = 1-cos(θ).

### Quantum Clustering (QC)

The Quantum Clustering (QC) algorithm [27] begins with a Parzen window approach, assigning a Gaussian of width σ to each data-point, thereby constructing *Ψ(x)*, where
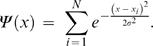

*Ψ(x)* can serve as a probability density that could have generated the data. Assuming this function to be the ground-state (lowest eigenvalue) of the Hamiltonian H of the Schrödinger equation:
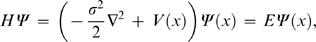
one can solve for the potential energy V uniquely, determining E through the condition that min(V(x)) = 0. The Schrödinger equation can be understood as a model balancing a clustering force (represented by the potential V) and a dispersive force (the second derivative term), that it is responsible for the fact that the data are not concentrated at the minima of V (bottoms of the potential energy).

An example of V(x) is shown in Fig. 1 for a dataset that comprises 798 genes. The classification of the genes into phases of the cell-cycle is illustrated by the different colors. The original data are given in 72 dimensions (time points). SVD is used to reduce them to two dimensions. The x-axis of this figure corresponds to cos(θ) of each of the 2D vectors representing the genes. As Fig.1 displays a cyclic trend is well observed. In conventional QC one would cluster the instances according to the valleys of V that they belong to. In TDQC we separate the data into two sets, α and β, where set α is defined by the deepest valley of V. To each dataset we reapply preprocessing, QC and division in a recursive manner. The stopping criterion of the recursion is when a subset contains no more than 2 data points. It is noteworthy that although SVD preprocessing is not a mandatory step, according to our experience, this routine is found very effective in both improving the clustering results and in significantly reducing the algorithm's runtime.

### ‘Glocal’ Hierarchical Approach: considering global information in bottom-up clustering

Data may come in two possible representations: *(1)* Feature space (a [*nXm*] matrix): each instance is measured according to its features (or attributes). Examples are: Gene expression, 3D coordinates of protein structures. *(2)* Distances or similarities (a [*nXn*] matrix): each instance is presented by its distance or similarity to another instance. Examples are: BLAST or Smith-Waterman matrices in proteomics. This representation leads to a square, and often, symmetric matrix.

**Figure 2:**
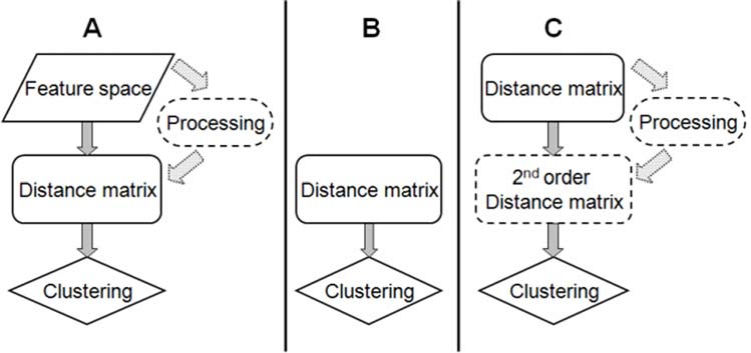
Three possible ways to handle data for generalized BU clustering. (A). Standard workflow when data are presented in feature space. (B). Standard workflow when only the distance matrix is known. (C). Our ‘glocal’ algorithm manipulates the distance matrix by using feature-space methods. Light gray arrows denote optional steps and dotted frames denote global consideration, such as SVD or PCA manipulations.

Clearly, the second representation is less informative than the first. It can be calculated from the first but, given only the distances, feature space cannot be reconstructed (except approximately as in Multidimensional Scaling [28]), as shown in Fig. 2 (A, and B). Standard BU relies on distances only, even when the data are given in feature space (e.g., in gene-expression analysis): distances are first derived and iterative lineage is performed on them[6].

In the cases where data is represented only by distances (Fig 2B), we argue that considering only the ‘nearest neighbors’ as the standard BU algorithms suggest, might end up neglecting relevant information in the data. We therefore suggest adding a global perspective to local clustering, namely glocal (global-local) clustering. This may be achieved by treating the distance matrix as an instance-by-feature matrix, i.e. using the instances as defining feature-space, after which BU is applied (Fig 2C). The intuitive idea behind this step is that the second order operation shifts positions of data points in a fashion that depends on the previous positions of their neighboring data points, while considering the distant points as well. This change causes dense groups to become denser, thus making it easier to form clusters. Another advantage of the glocal approach is that the instance-by-feature matrix allows one also to apply processing routines (e.g., SVD, PCA) to achieve dimensionality reduction before applying the clustering algorithm (see, e.g., [16]).

### Statistical Criteria for Classification Quality

A clear limitation of hierarchical clustering (whether TD or BU) is the inherent difficulty in the evaluation scheme. Jain & Dubes argue that the hierarchy of clustering can be evaluated only when an expert-hierarchy is available (we use the term ‘expert’ to describe the external data labeling [3]). Quite often such expert-hierarchies are unavailable and no gold standard criterion exists [13]. Alternative measures that do not capture the hierarcy per-se have been suggested [29].

We address the instances where expert-classification of data is provided, and combine 3 assessment methods to describe different qualities of the clustering tree.

1. **Node Score** Since each node specifies a cluster, enrichment *p*-values can be calculated to assign the given node with one of the classes in the data. This is done by using the hypergeometric probability density function. The significance *p*-value of observing *k* instances assigned by the algorithm to a given category in a set of *n* instances is given by 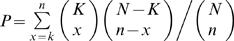, where *K* is the total number of instances assigned to the class (the category) and *N* is the number of instances in the dataset. The *p*-values for all nodes and all classes may be viewed as dependent set estimations; hence we apply the False Discovery Rate (FDR) criterion to them requiring *q<0.05* [30]. *P-*values that do not pass this criterion are considered non-significant. We further apply another conservative criterion; namely, a node is considered significant only if *k≥n/2* (i.e., the majority of its instances belongs to the enriched category).
2. **Level Score** A level *l* of the tree contains all nodes that are separated by *l* edges from the root, i.e., that share the same Breadth First Search (BFS) mapping. Each level specifies a partition of the data into clusters. Choosing for each node, the class for which it turned out to have a significant node score, we evaluate its Jaccard-score *(J = tp/(tp+fn+fp)*, where *tp* is the number of true positive cases, *fn* the number of false negative cases and *fp* the number of false positive cases). If the node in question has been judged to be non-significant by the enrichment criterion, its J-score is set to null. The level score is defined as the average of all J-scores at the given level.
3. **Tree Score** We define the weighted best-J-Score (
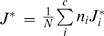
) where J^*^_i_ is the best J-Score for class *i* in the tree, *n_i_* is the number of instances in class *i*, *c* is the number of classes and *N* is the number of instances in the dataset. This criterion provides a single number specifying the quality of the tree based on a few nodes that contain optimal clusters. This score or its close variation has been applied to measure the quality of proteins families [31] and document classification [12], [32].

## Results

All datasets were analyzed using two nonhierarchical algorithms, QC and K-Means, several variants of Bottom-Up algorithms, single-linked (BU-S), average-linked (BU-A) and complete-linked (BU-C) [3], [4] and two Top-Down algorithms, PDDP and our TDQC.

The results of the hierarchical algorithms were evaluated using a combination of the 3 scoring methods presented above as follows. *(A)* The node-score, the clustering tree is presented with its enrichment markers for every tree node. It combines a qualitative and graphical description of the results. Recall that the graphical description is presented for visualization purposes only. *(B)* The level-score, the average J-score of each level in the tree, which provides both qualitative and quantitative information on the algorithm's performance along the hierarchy. *(C)* The tree score, the weighted best J-scores. Being a single score, the tree score provides a criterion for comparison of hierarchical algorithms to algorithms that are nonhierarchical in nature.

**Figure 3:**
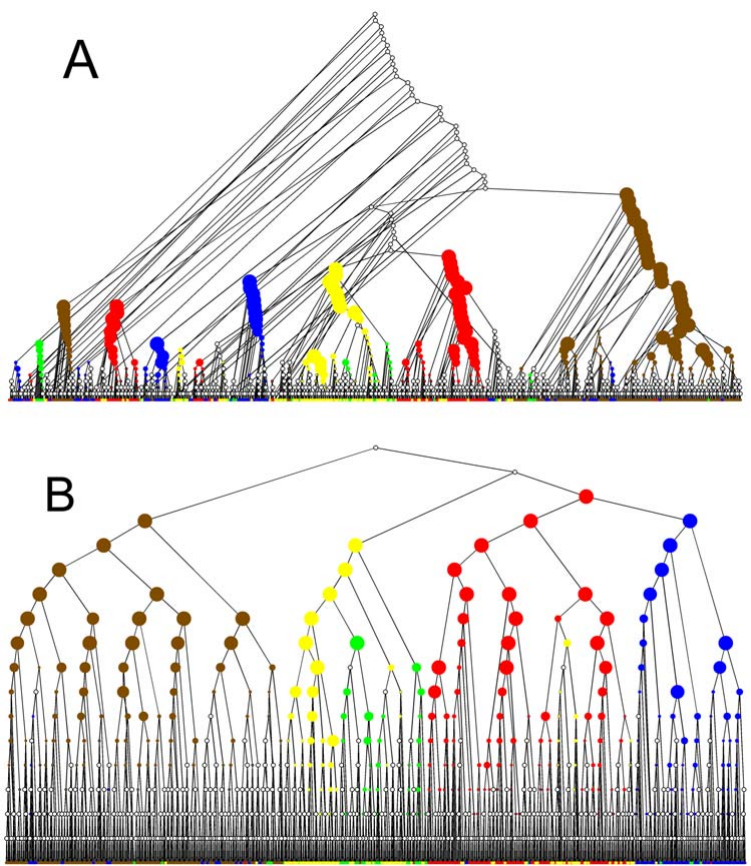
Hierarchical trees of the 798 cell cycle genes for BU-A (A) and TDQC (B) algorithms. Color codes specify the five cell cycle classes as in (Fig. 1). Dot sizes indicate statistical enrichment levels (larger sizes correspond to smaller p-values). Uncolored nodes represent non-significant enrichment.

Fig. 3 A, B displays the trees as generated by a BU-A algorithm (using Euclidean metric and average linkage), and the TDQC algorithm when applied to the Cell Cycle dataset. Note that the BU-A performed best out of all the BU variants (Table 1).

**Table 1:** Clustering scores (tree score) of nonhierarchical (QC, K-Means) and hierarchical algorithms.

	Elements	Features	Classes	Non-hierarchical		Hierarchical				
				QC	K-Means	BU			TD	
						BU-S	BU-A	BU-C	PDDP	TDQC
**Cell cycle**	798	72	5	0.613	0.537 (0.06)	0.265	0.472	0.409	0.542	**0.646**
**Leukemia**	72	7129	4	0.758	0.519 (0.1)	0.465	0.522	0.53	0.545	**0.804**
**S&P**	487	273	10	0.400	0.306 (0.05)	0.2	0.261	0.445	0.441	**0.504**

K-Mean was performed 10 times and averaged (and std is in parenthesis), Hierarchical algorithms are BU (S, A and C marks the Single, Average, Complete, respectively) and TD (PDDP, TDQC) algorithms. Best scores are bold faced.

Some prominent patterns emerge from Fig. 3 A, B and almost identical conclusions can be drawn from all other datasets: *1.* The BU tree is far more unbalanced relative to the TD tree. *2.* The TD algorithm performs best on higher levels of the tree, whereas the BU algorithm performs better on lower levels of the tree. This can be seen here by observing where the statistical enrichment of nodes is highest. *3.* TD clusters (sub-trees) are very coherent, i.e. it is very rare for significant nodes of one color to have children of another color.

**Figure 4:**
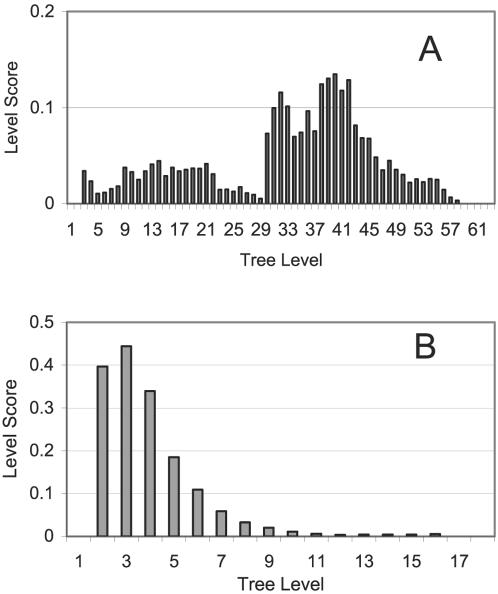
Level scores of (A) BU-A and (B) TDQC for the cell cycle dataset. Tree levels are counted from the root. Note the different scale for the Y-axes.

Next we turn to measuring clustering quality by comparing level-scores in Fig 4. The TDQC algorithm has a high maximal score (0.44) and displays an almost monotonic decrease with increasing tree-level. The BU algorithm exhibits significantly different behavior. Namely, it leads to a bimodal distribution and its much smaller (0.13) maximal score is located at low hierarchy levels.

**Figure 5:**
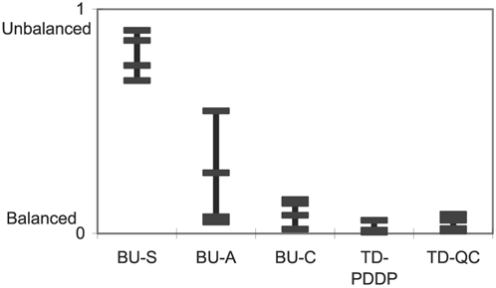
The relative depths of the trees generated by the various algorithms when applied to 4 gene expression, stock market and protein family.

The two trees also differ in their tree depth. The depth of the tree (*D*) is defined as the distance between the root and the farthest leaf. A completely balanced (binary) tree with *N* nodes is *log_2_(N)* deep whereas a completely unbalanced tree is *N* deep. Figure 5 displays the relative depths (*(D- log_2_(N))/(N- log_2_(N))*) of all trees generated by different BU and TD algorithms when applying them to the 4 datasets presented in this study.

Despite the fact that each of the datasets used in this study comprises a different number of instances and is differently represented (e.g., similarity, raw data), we observe common trends in Fig. 5 and conclude that the nature of the algorithm governs the shape of the tree. TD algorithms tend to generate more balanced trees, and as a result have fewer levels (in the PDDP algorithm each binary division is essentially into sub-clusters of equal sizes); BU algorithms usually generate deeper trees where single-linked (BU-S) algorithms tend to produce chain-like trees, whereas complete-linked algorithms (BU-C) create more balanced trees [33]

Finally we turn to the global measures of clustering quality, based on comparisons with expert classifications. Table 1 summarizes the tree scores of all algorithms when applied to the gene-expression and the stock-market benchmarks. The TD algorithms outperform the BUs in all these cases. This is presumably due to the fact that the expert classifications represent global partitions of the data, whereas the BU approaches are fairly poor (BU-S in particular [1]. TDQC outperforms all other algorithms, including the nonhierarchical QC.

### Evaluation of Different Granularity Levels in Protein Sets

In order to expand our analysis on data that are inherently hierarchical, we analyzed a set of proteins associated with annotation of channels. This set comprises well-studied proteins to which functional annotations are assigned based on experimental evidence and evolutionary homology relationships [34]. Our set is composed of proteins associated with ‘ion channel activity’, which form a subset of proteins belonging to ‘transporters and channels’ (Gene Ontology ID-6811). These are membranous proteins that function in the directional translocation of substances across membranes. The directional translocation is between cell compartments and between cells and the environment. These proteins are defined by InterPro experts as belonging to 3 classes according to their gating mode: ligand-gated, voltage-gated and ‘others’. The last group includes proteins that are gated by nucleotides (e.g., as in the case of the cystic fibrosis chloride channel) and several channels that have a mixed gating mode or yet undefined properties. This ‘gating mode’ property dominates other characteristics of the channels and receptors including their multimeric nature, the number and nature of their accessory subunits, the number of transmembrane domains, etc. These 3 classes are further divided into other granularity levels of 11 and 19 classes respectively (see Methods).

We tested the various clustering algorithms to see how well they met the different granularity levels (Table 2). This served to show which approaches are appropriate at different granularity levels.

**Table 2:** Clustering scores of different algorithms applied to the ion channel proteins.

Classes	Non-hierarchical		Hierarchical				
			BU			TD	
	QC	K-Means	BU-S	BU-A	BU-C	PDDP	TDQC
**3**	0.6859	0.565 (0.13)	0.613	0.395	0.382	0.771	**0.808**
**11**	0.4626	0.533 (0.05)	0.338	0.34	0.245	0.567	**0.61**
**19**	0.3218	0.515 (0.06)	0.23	0.32	0.268	0.64	**0.655**

Scores are measured according to the appropriate granularity level (for 3, 11 and 19 classes).

Clearly, comparing the performance of the different algorithms for different granularity levels (Table 2) shows the inferior performance of the BU algorithms. To address the question of suitability of the algorithm to the data, we compared the best TD to the best BU algorithms (TDQC and BU-A, respectively). Since the BU level-scores have a bimodal pattern (as in Fig 4) with maxima occurring in the 1^st^ and 4^th^ quartiles, we compared the maxima of the level scores of the two algorithms in these two quartiles.

**Figure 6:**
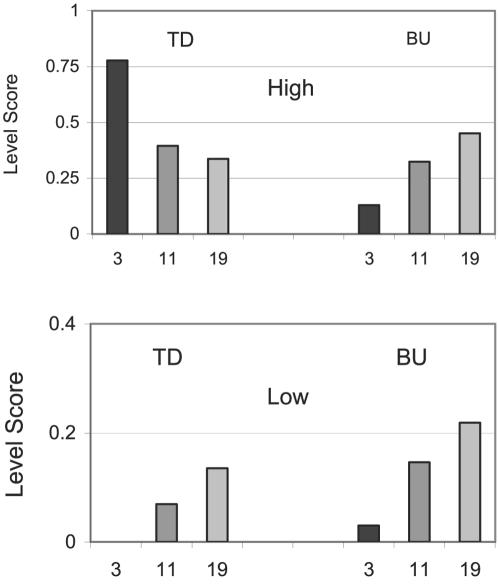
Comparing the two extreme parts of the level scores for the TDQC (left), and BU-A (right) algorithms for different levels of granularity (3, 11 and 19 classes). ‘High’ and ‘Low’ refer to the 1^st^ and 4^th^ quartiles for the levels in the resulting trees. Note the different scale for the Y-axes.

As depicted in Fig 6, the results show that in the high levels of the tree, the TD algorithm outperforms the BU. The performance of the TD algorithm declines when the granularity from 3 to 19 is increased, whereas the BU performance only improves gradually. At the other end of the scale (low levels of the trees), the scores of both methods improve when granularity increases. However, at all granularity levels, the BU algorithm outperforms TD. Note that in both methods, the overall performance is rather poor for the 4^th^ quartiles of the trees (level score<0.22). For the 1^st^ quartile, the score of the TDQC reaches 0.78. Similar conclusions were obtained when applying different scoring methods, such as counting the significant nodes in each level (not shown).

Since the high levels reflect a global view of the data whereas low levels account for local aspects, TD algorithms appear to be more appropriate in describing the high level patterns, whereas the opposite holds for local patterns of the data.

### Glocal clustering improves the quality of BU algorithms

**Figure 7:**
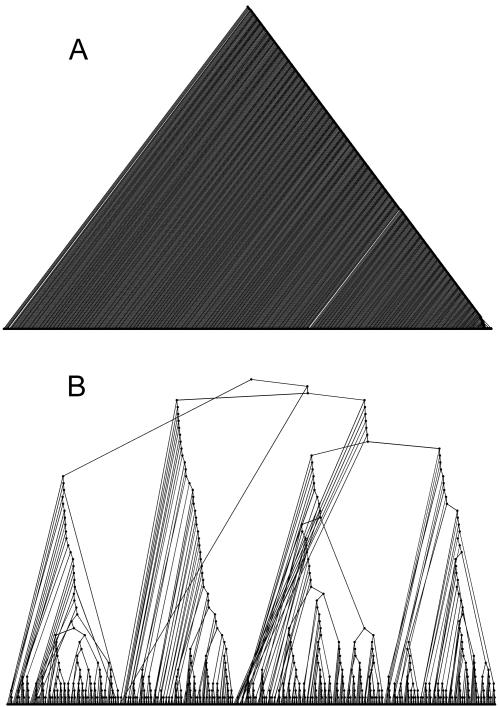
The hierarchical tree of the BU (A) and glocal (B) algorithms, as applied to the Ion channel dataset. Single linkage wad used in both algorithms.

In the Ion Channel dataset, the instances (proteins) are represented by their distance from each other (E-value). Following the standard BU approach involves jointing sub-clusters solely according to their mutual distance. As suggested above (see Methods), we argue that considering the distances of all sub-clusters in the clusters-merging process may improve the clustering quality. We therefore applied the glocal protocol to the dataset and compared its results with the standard BU algorithms. Fig. 7 displays the trees as generated by the BU (A) and Glocal (B) algorithms. In this case both methods use Euclidian distance and single linkage; similar trend was observed in other combinations.

**Figure 8:**
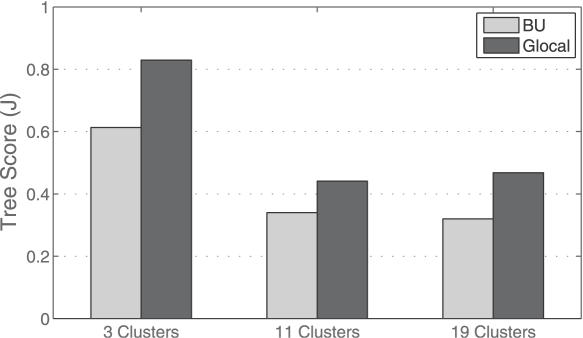
Tree scores of BU and glocal algorithms for different levels of granularity (3, 11 and 19 classes). Shown are the best results for each approach (single, average or complete).

As Fig 7 shows, the glocal tree is more balanced than the BU tree. Moreover, three clusters are well observed in the glocal tree, while no apparent partition is detected in the BU tree. As the two trees display significantly different structures, we turned to evaluate how well they capture the expert classification at the three resolution levels. Fig. 8 displays the tree scores of both algorithms, given the 3 granulation levels.

As displayed in Fig. 8, the glocal protocol improved the clustering results at all granulation levels. The tree, as generated in this way is also more balanced and more informative. Overall, in many other datasets (not shown), we found that adopting this very simple approach may significantly improve clustering results, when comparing to the standard BU implementation.

### Biological Interpretations Based on TDQC

**Figure 9:**
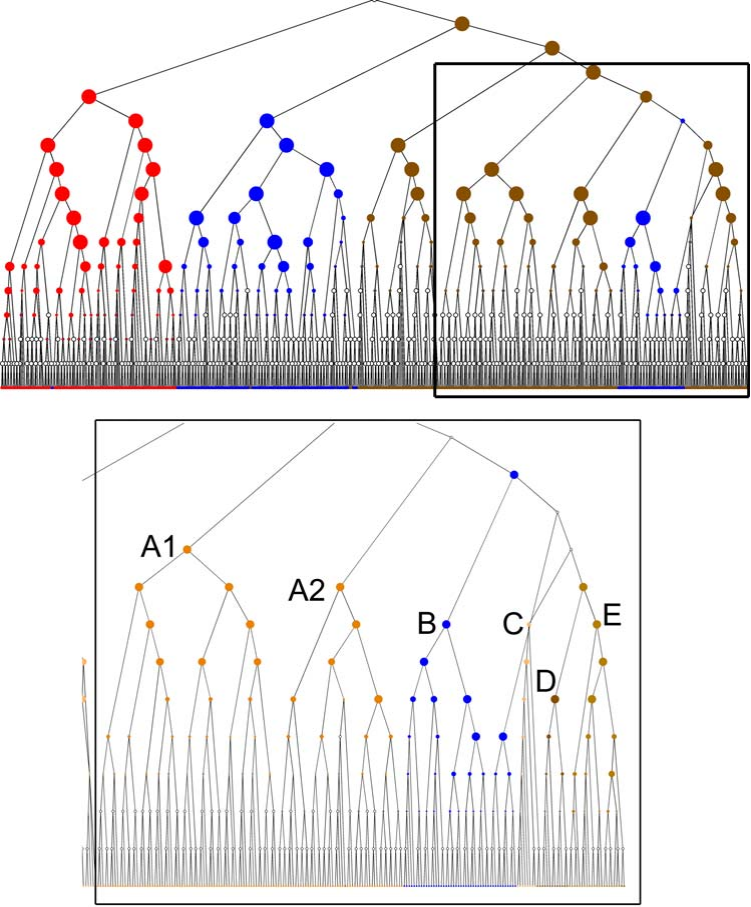
Hierarchical tree produced by the TDQC algorithm for 518 proteins of ion channels. Red, blue and brown are assigned to the 3 classes: “others”, “ligand-gated” and “voltage-gated”, respectively. The bottom inset is a zoom of a subset of the tree marked by the frame and according to level of granularity of 11 classes. Sub-trees are all indicated in brown and marked by their identity. A1, A2 - K+ channels ; B – NMDA and Kianate receptors (35 proteins); C – Ryanodine receptors (10 – proteins); D – Na+/H+ exchangers (11 proteins); E – TRP channels (18 proteins). A1 and A2 are separate branches with A1 (73 proteins) including all Kv channels, and A2 with the Cyclic nucleotide-gated channel (51 proteins). Recall, that the top and bottom panels show the same tree.

With the rapid expansion of available biological data, the reference to an ‘expert’ often means there has been a combination of automatic and manual efforts. The automatic TDQC algorithm was very successful (score of 0.808) in classifying the coarse granularity of the 518 proteins into 3 classes (Table 2). Nevertheless, the algorithm can also reveal partitions of the data overlooked by these experts (Fig 9). It can be seen in the graph that a group of 35 proteins marked as ‘others’ is embedded within the sub-tree of ‘voltage gated channels’ (blue nodes within a brown sub-tree). Inspecting this set of 35 proteins indicates that they are composed of 2 functionally different glutamate ionotropic receptors belonging to NMDA (19 proteins) and Kainate (12 proteins) families (known as NR1-2 and GluR5-7, respectively). For an additional 4 proteins in this set, no clear assignment is provided. Interestingly, an additional set of ionotrophic glutamate receptors set known as AMPA (with 12 proteins, GluR1-4) are separated from the NMDA-Kainate group. Thus, the TDQC partitioned the AMPA ionotrophic glutamate receptors separately from the Kainate and NMDA. Other properties of these receptors including their selectivity, multimeric structures and evolutionary relatedness indeed favor the partition of the AMPA receptors away from the Kinate-NMDA [35]. In high quality annotation systems (such as Pfam, SMART and the InterPro integration system) no such separation appeared.

We further investigated the relationship between the various subtypes of voltage gated channels (marked in brown, Fig. 9) by using a finer granularity of 11 classes (Tables S1, S2 supplement). A clear partition was generated by the TDQC and the Kainate-NMDA set (Fig 9, bottom, marked B). This set is more closely related to the C and D clusters than to A1 and A2. All proteins in cluster A2 are voltage-gated K^+^ channels that belong to the Kv1 superfamily and the cyclic regulated channels (whereas the proteins in A1 are Kv1-Kv11). The C cluster comprises a group of 18 TRP channels. All TRP channels are permeable to cations. Although only 2 of the channels (TRPM4 and TRPM5) are impermeable to Ca^2+^, 2 others (TRPV5 and TRPV6) are highly Ca^2+^ permeable [36]. Cluster D includes Ryanodine and Inositol 1, 4, 5-trisphosphate (IP3) receptors that are intracellular Ca^2+^ release channels [37]. Cluster E represents a class of Na^+^/H^+^ exchangers [38]. Thus the close relationship of the NMDA-Kainate group to Ca^2+^ channels (in clusters C and D) supports their functional relevance and the shared mode of their regulation. Thus, TDQC provides a tree- like structure that not only captures the expert partition but exposes additional connectivity that was overlooked. This group of channels is of special interest as they are targets for pharmaceutical strategies in neurodegenerative diseases and mental pathologies. Their functional partition is far richer than that reflected by their ion conductance properties [39].

## Discussion

We carried out a comparative analysis of five hierarchical clustering algorithms and two nonhierarchical ones, applying them to different types of datasets from various sources. We showed that TD algorithms are consistently superior to BU and nonhierarchical algorithms. In particular, TDQC was found to outperform both TD and BU state-of-the-art algorithms. This applies to data from gene expression, protein families and the stock market.

BU algorithms have some advantages in identifying local relations in the data whereas TD methods capture global patterns. When general patterns are sought, as is the often the case in preliminary stages of data analysis, conventional BU clustering methods should be avoided and replaced by TDs. The latter result in more balanced trees and may be halted – if desired – well before generating the entire tree.

When the data are provided as similarities or distances between instances, we find that a simple manipulation based on all relationships within the data (all distances), may significantly improve the clustering results of the BU approach. This glocal algorithm imposes some global information on the BU making it more competitive with TD algorithms. In summary, global approaches in the exploratory process of clustering, in particular TD or glocal algorithms, are strategies that should not be overlooked.

Although there are ongoing efforts to establish expert hierarchies in various domains, these attempts are riddled with difficulties. High level annotations, often manually catalogued (e.g., GO, UniProt keywords in proteomics) are strongly biased by current knowledge. As a result, that part of the data (in, e.g., protein families) that has been thoroughly studied may possess a rich tree-structure whereas the rest is poorly mapped and weakly annotated. Applying unsupervised methods, such as the TD clustering methods presented here, can leverage the quality of these manually-created mappings. As demonstrated, it can also provide insights into areas that have been missed and correct erroneous annotations.

ClustTree, a graphical Matlab toolbox for applying various hierarchical clustering algorithms and testing their quality is provided and freely available at http://adios.tau.ac.il/clustree/ or http://www.protonet.cs.huji.ac.il/clustree (alternative).

## Supporting Information

Table S1 Keywords and classes size nesting of the ion-channel group (GOID5216)
(0.04 MB PDF)

Table S2 Tables (A) and (B): InterPro Classifications of the ion-channel group (GOID5216).
(0.04 MB PDF)

Table S3 Cell Cycle Dataset: Classes information.
(0.01 MB PDF)

Table S4 Leukemia Dataset: Classes information.
(0.02 MB PDF)

Table S5 S&P Dataset: Classes information.
(0.01 MB PDF)

## References

[1] D'Haeseleer P (2005). How does gene expression clustering work?. Nat Biotechnol.

[2] Eisen MB, Spellman PT, Brown PO, Botstein D (1998). Cluster analysis and display of genome-wide expression patterns.. PNAS.

[3] Jain AK, Dubes RC (1988). Algorithms for Clustering Data..

[4] Duda RO, Hart PE, Stork DG (2000). Pattern Classification: Wiley-Interscience..

[5] Handl J, Knowles J, Kell DB (2005). Computational cluster validation in post-genomic data analysis.. Bioinformatics.

[6] Planet PJ, DeSalle R, Siddall M, Bael T, Sarkar IN (2001). Systematic Analysis of DNA Microarray Data: Ordering and Interpreting Patterns of Gene Expression.. Genome Res.

[7] Rune M (2007). Methods, algorithms and tools in computational proteomics: A practical point of view.. PROTEOMICS.

[8] Apweiler R, Biswas M, Fleischmann W, Kanapin A, Karavidopoulou Y (2001). Proteome Analysis Database: online application of InterPro and CluSTr for the functional classification of proteins in whole genomes.. Nucleic Acids Res.

[9] Sasson O, Vaaknin A, Fleischer H, Portugaly E, Bilu Y (2003). ProtoNet: hierarchical classification of the protein space.. Nucleic Acids Res.

[10] Chipman H, Tibshirani R (2006). Hybrid hierarchical clustering with applications to microarray data.. Biostat.

[11] MathWorld (2007). Matlab Statistics Toolbox. 6.1 ed: MathWorld..

[12] Steinbach M, Karypis G, Kumar V (2000). A comparison of document clustering techniques; Boston..

[13] Cimiano P, Hotho A, Staab S (2004). Comparing conceptual, divisive and agglomerative clustering for learning taxonomies from text; pp. 435–439..

[14] Boley D (1998). Principal Direction Divisive Partitioning; 1998. pp. 325–344..

[15] Savaresi MS, Boley D (2004). A comparative analysis on the bisecting K-means and the PDDP clustering algorithms.. Intelligent Data Analysis.

[16] Varshavsky R, Linial M, Horn D (2005). COMPACT: A Comparative Package for Clustering Assessment..

[17] Golub TR, Slonim DK, Tamayo P, Huard C, Gaasenbeek M (1999). Molecular Classification of Cancer: Class Discovery and Class Prediction by Gene Expression Monitoring.. Science.

[18] Sharan R, Shamir R CLICK: A Clustering Algorithm with Applications to Gene Expression Analysis..

[19] Getz G, Levine E, Domany E (2000). Coupled two-way clustering analysis of gene microarray data.. PNAS.

[20] Slonim N, Atwal GS, Tkacik G, Bialek W (2005). Information-based clustering.. PNAS.

[21] Altschul S, Madden T, Schaffer A, Zhang J, Zhang Z (1997). Gapped BLAST and PSI-BLAST: a new generation of protein database search programs.. Nucl Acids Res.

[22] Alter O, Brown PO, Botstein D (2000). Singular value decomposition for genome-wide expression data processing and modeling.. PNAS.

[23] Horn D, Axel I (2003). Novel clustering algorithm for microarray expression data in a truncated SVD space.. Bioinformatics.

[24] Cangelosi R, Goriely A (2007). Component retention in principal component analysis with application to cDNA microarray data.. Biology Direct.

[25] Varshavsky R, Horn D, Linial M (2007). Clustering Algorithms Optimizer: A Framework for Large Datasets..

[26] Landauer TK, Foltz PW, Laham D (1998). Introduction to Latent Semantic Analysis.. Discourse Processes.

[27] Horn D, Gottlieb A (2002). Algorithm for data clustering in pattern recognition problems based on quantum mechanics.. Physical Review Letters.

[28] Kruskal JB, Wish M (1981). Multidimensional scaling..

[29] Torrente A, Kapushesky M, Brazma A (2005). A new algorithm for comparing and visualizing relationships between hierarchical and flat gene expression data clusterings.. Bioinformatics.

[30] Benjamini Y, Hochberg Y (1995). Controlling the False Discovery Rate: A Practical and Powerful Approach to Multiple Testing.. Journal of the Royal Statistical Society Series B (Methodological).

[31] Kaplan N, Friedlich M, Fromer M, Linial M (2004). A functional hierarchical organization of the protein sequence space.. BMC Bioinformatics.

[32] Zhao Y, Karypis G (2002). Evaluation of hierarchical clustering algorithms for document datasets..

[33] Hansen P, Delattre M (1978). Complete-link cluster analysis by graph coloring.. J American Stat Ass.

[34] Ren Q, Paulsen IT (2005). Comparative Analyses of Fundamental Differences in Membrane Transport Capabilities in Prokaryotes and Eukaryotes.. PLoS Computational Biology.

[35] Zorumski CF, Thio LL (1992). Properties of vertebrate glutamate receptors: Calcium mobilization and desensitization.. Progress in Neurobiology.

[36] Owsianik G, Talavera K, Voets T, Nilius B (2006). PERMEATION AND SELECTIVITY OF TRP CHANNELS.. Annual Review of Physiology.

[37] Berridge M (2004). Conformational Coupling: A Physiological Calcium Entry Mechanism. Sci STKE 2004: pe33-..

[38] Orlowski J, Grinstein S (2004). Diversity of the mammalian sodium/proton exchanger SLC9 gene family.. PflÃ¼gers Archiv European Journal of Physiology.

[39] Kaczmarek LK (2006). Non-conducting functions of voltage-gated ion channels..

