# Supplementary material for: Global Considerations in Hierarchical Clustering Reveal Meaningful Patterns in Data
Source: PLoS One. 2008 May 21;3(5):e2247. doi: 10.1371/journal.pone.0002247 (PMC2375056; doi:10.1371/journal.pone.0002247)
Supplement: Table S1 — Keywords and classes size nesting of the ion-channel group (GOID5216) (0.04 MB PDF) [file pone.0002247.s001.pdf]

Keyword (3)	Keyword (11)	Keyword (19)	# elements (3)	# elements (11)	# elements (19)	IPR Keywords
Ion transport protein			**233**			5821
	Na+ Related channels			36		1696, 1873, 1953, 1118,2090
		Na+ channel			*16*	1696
		Na+ channel, amiloride-sensitive			*9*	1873
		Na+/H+ exchanger, isoform 2 (NHE2)			*4*	1653
		Na+/H+ exchanger, isoform 3 (NHE3)			*6*	1118
		Na+/H+ exchanger, isoform 6 (NHE6)			*1*	2090
	Potassium			123		3968, 3938
		Kv channel			*61*	3968
		EAG/ELK/ERG potassium channel			*50*	3938
		KCNQ voltage-gated potassium channel			*12*	3937
	Ca2+ channel, alpha subunit			32	32	2077
	Intracellular calcium-release channel			16	16	699
	Transient receptor potential protein			26	26	2153
Neurotransmitter-gated ion-channel			**119**			6201
	Gamma-*aminobutyric-acid A receptor,* alpha *& beta subunit*			37		*2289*,1390
		Gamma-aminobutyric-acid A receptor, beta *subunit*			*25*	1390
		Gamma-aminobutyric-acid A receptor, alpha *subunit*			*12*	*2289*
		Nicotinic acetylcholine receptor		82	82	2394
Other			**166**			1185, 1320, 272, 2667, 1425
	Large-conductance mechanosensitive channel			19	19	1185
	Ionotropic glutamate receptor			47	47	1320
	FXYD protein			26	26	272
	Isopentenyl-diphosphate delta-isomerase			44	44	2667
	Bacterial rhodopsin			30	30	1425
**TOTAL**			**518**	**518**	**518**	

Table 1: Keywords and classes size nesting of the ion-channel group (GOID:5216)
