# Supplementary material for: Global Considerations in Hierarchical Clustering Reveal Meaningful Patterns in Data
Source: PLoS One. 2008 May 21;3(5):e2247. doi: 10.1371/journal.pone.0002247 (PMC2375056; doi:10.1371/journal.pone.0002247)
Supplement: Table S2 — Tables (A) and (B): InterPro Classifications of the ion-channel group (GOID5216). (0.04 MB PDF) [file pone.0002247.s002.pdf]

Cluster	Name	# of elements	IPR
1	Ion transport related	233	1696, 1873, 1953, 1118,2090, 3968, 3938, 3937,2077, 699,2153
2	Ligand related	119	1390,*2289,* 2394
3	Other	166	1185,1320,272,2667,1425

Table 2a – InterPro Classification to 3 classes

Cluster	Name	# of elements	IPR
1	Na+ channel	36	1696, 1873, 1953, 1118, 2090
2	Potassium channel	123	3968, 3938, 3937
3	Ca2+ channel, Related	48	2077, 699
4	Transient receptor potential protein	26	2153
5	*Gamma-aminobutyric-acid A receptor,* beta, alpha *subunits*	37	1390,*2289*
6	Nicotinic acetylcholine receptor	82	2394
7	Large-conductance mechanosensitive channel	19	1185
8	Ionotropic glutamate receptor	47	1320
9	FXYD protein	26	272
10	Isopentenyl-diphosphate delta-isomerase	44	2667
11	Bacterial rhodopsin	30	1425

Table 2b – InterPro Classification to 11 classes

Cluster	Name	# of elements	IPR
1	Na+ channels	16	1696
2	Na+ channel, amiloride-sensitive	9	1873
3	Na+/H+ exchanger, isoform 2 (NHE2)	4	1653
4	Na+/H+ exchanger, isoform 3 (NHE3)	6	1118
5	Na+/H+ exchanger, isoform 6 (NHE6)	1	2090
6	Kv channel	61	3968
7	EAG/ELK/ERG potassium channel	50	3938
8	KCNQ voltage-gated potassium channel	12	3937
9	Ca2+ channel, alpha subunit	32	2077
10	Intracellular calcium-release channel	16	699
11	Transient receptor potential protein	26	2153
12	*Gamma-aminobutyric-acid A receptor,* beta *subunit*	25	1390
13	*Gamma-aminobutyric-acid A receptor,* alpha *subunit*	12	*2289*
14	Nicotinic acetylcholine receptor	82	2394
15	Large-conductance mechanosensitive channel	19	1185
16	Ionotropic glutamate receptor	47	1320
17	FXYD protein	26	272
18	Isopentenyl-diphosphate delta-isomerase	44	2667
19	Bacterial rhodopsin	30	1425

Table 2c – InterPro Classification to 19 classes
