# Supplementary material for: Global Considerations in Hierarchical Clustering Reveal Meaningful Patterns in Data
Source: PLoS One. 2008 May 21;3(5):e2247. doi: 10.1371/journal.pone.0002247 (PMC2375056; doi:10.1371/journal.pone.0002247)
Supplement: Table S3 — Cell Cycle Dataset: Classes information. (0.01 MB PDF) [file pone.0002247.s003.pdf]

Class	Number of elements	Color in figures
G1	299	brown
S	70	green
S/G2	121	yellow
G2/M	195	red
M/G1	113	blue

Table 3: Cell Cycle Dataset: Classes information
