# Supplementary material for: Global Considerations in Hierarchical Clustering Reveal Meaningful Patterns in Data
Source: PLoS One. 2008 May 21;3(5):e2247. doi: 10.1371/journal.pone.0002247 (PMC2375056; doi:10.1371/journal.pone.0002247)
Supplement: Table S4 — Leukemia Dataset: Classes information. (0.02 MB PDF) [file pone.0002247.s004.pdf]

Class	Number of elements	Color in figures
T-cell	38	brown
B-cell	9	red
AML treat	15	blue
AML no treat	10	yellow

Table 4: Leukemia Dataset: Classes information
