# Supplementary material for: Global Considerations in Hierarchical Clustering Reveal Meaningful Patterns in Data
Source: PLoS One. 2008 May 21;3(5):e2247. doi: 10.1371/journal.pone.0002247 (PMC2375056; doi:10.1371/journal.pone.0002247)
Supplement: Table S5 — S&P Dataset: Classes information. (0.01 MB PDF) [file pone.0002247.s005.pdf]

**Class**	**Number of elements**	**Color in figures**
Energy	23	Brown
Materials	33	Red
Industrials	58	Blue
Consumer Discretionary	83	Green
Consumer Staples	35	Yellow
Health Care	47	Magenta
Financials	80	Cyan
Information Technology	81	Gray
Telecommunication Services	11	Purple
Utilities	36	Olive

Table 5: S&P Dataset: Classes information
